# New Onset of Dermatomyositis/Polymyositis during Anti-TNF-****α**** Therapies: A Systematic Literature Review

**DOI:** 10.1155/2014/179180

**Published:** 2014-01-29

**Authors:** Alexandra Maria Giovanna Brunasso, Werner Aberer, Cesare Massone

**Affiliations:** ^1^Department of Dermatology, Medical University of Graz, Graz, Austria; ^2^Department of Dermatology and Venereology, Medical University of Graz, Auenbruggerplatz 8, 8036 Graz, Austria

## Abstract

We performed a systematic search of databases from 1990 to 2013 to identify articles concerning the new onset of dermatomyositis/polymyositis (DM/PM) in patients treated with anti-TNF-**α** therapy. We retrieved 13 publications describing 20 patients where the new onset of DM/PM after anti-TNF-**α** therapy was recorded. 17 patients were affected by rheumatoid arthritis (RA), one by Crohn's disease, one by ankylosing spondilytis, and one by seronegative arthritis. In 91% of the cases antinuclear autoantibodies were detected after the introduction of anti-TNF-**α** therapy. In 6 patients antisynthetase antibodies were detected and other clinical findings as interstitial lung disease (ILD) were recorded. Improvement of DM/PM after anti-TNF suspension (with the concomitant use of other immunosuppressors) was recorded in 94% of cases. The emergence of DM/PM and antisynthetase syndrome seem to be associated with the use of anti-TNF-**α** agents, especially in patients with chronic inflammatory diseases (mainly RA) with positive autoantibodies before therapy initiation. In particular, physicians should pay attention to patients affected by RA with positive antisynthetase antibodies and/or history of ILD. In those cases, the use of the TNF-**α** blocking agents may trigger the onset of PM/DM or antisynthetase syndrome or may aggravate/trigger the lung disease.

## 1. Introduction

Dermatomyositis (DM) is a chronic, idiopathic inflammatory myopathy, potentially life threatening, that affects individuals of all ages [1]. The estimated incidence of DM has been calculated as 9.63 per 1 million persons, with a prevalence of 21.42 per 100,000 persons [1]. DM and PM can be associated with other autoimmune and connective tissue diseases [1, 2]. Polymyositis (PM) is a rare, chronic, idiopathic inflammatory myopathy that affects individuals over the age of 20 years and is more common in women [1, 2]. The definitive diagnosis requires the exclusion of DM and other inflammatory myopathies [1, 2].

Raised levels of TNF-*α* have been demonstrated both in serum of patients with chronic DM and inside the calcium deposits (calcinosis cutis) [3]. It has also been reported that the soluble forms of the receptors TNF-R55 and TNF-R75 are increased in DM/PM sera [4]. The TNF-*α* allele, called TNF*α*-308A, and the linkage disequilibrium of the HLA-B locus have been associated with a higher risk of calcinosis, prolonged disease course, and ulcerative skin disease [5]. The polymorphism of the osteopontin promoter in conjunction with the TNF*α* 308A allele promotes high serum levels of interferon-*α* in untreated patients with DM of European ancestry [5]. Such patients usually present a more aggressive disease course and develop calcinosis [5]. The TNF*α*-308A allele by itself has also been associated with vascular occlusion and increased production levels of TNF-*α* [6]. The role of type-I interferon (IFN)-mediated innate immunity in DM and PM-affected patients seems to be crucial [4, 7]. The induction of INF-alpha can be the result of immune complexes containing anti-Ro or anti-Jo-1 antibodies and RNA that activate IFN-*α* production in plasmacytoid dendritic cells [8, 9]. In patients with DM and negative autoantibodies, the presence of MX-1 protein in capillaries suggests another cellular IFN-*α* source and induction mechanism [8, 9].

Biological agents, in particular TNF-*α* blocking agents, have been proposed as potential steroid-sparing agents and as long-term therapies in addition or substitution to corticosteroid therapy [10–12]. According to Martin et al., anti-TNF-*α* therapy has become the most commonly considered second- or third-line therapy for patients with refractory juvenile DM in the UK even in the absence of prospective randomized control trials (RCTs) to support such use [13]. Paradoxically, there are some reports in the literature regarding the new onset of DM/PM in patients affected by other diseases [as rheumatoid arthritis (RA), Crohn's disease, and so forth] during etanercept, infliximab, and adalimumab [14–25].

We therefore conducted an up-to-date systematic review regarding the new onset of DM/PM in patients treated with TNF-*α* blockers for different conditions and described the reports in regard to the patients characteristics and evaluated the role of autoantibodies, duration of therapy, and clinical picture when possible. We hope that these results will help physicians in their choices of patients with different conditions and those eligible to receive anti-TNF-*α* agents.

## 2. Methods

We performed a systematic search of databases (PubMed, Embase, Cochrane Central, and Web of Science) from January 1990 until July 2013, using the following keywords and [MESH FORMS]: “dermatomyositis”, and/or “polymyositis”, and/or “induced” and/or “tumor necrosis factor or antitumor necrosis factor alpha”, and/or “TNF”, and/or “etanercept”, and/or “lenercept”, and/or “infliximab”, and/or “adalimumab”, and/or “golimumab”, and/or “certolizumab”, and/or “polymyositis.” No exclusion criteria were applied, and only articles in English, Spanish, German, Italian, and Portuguese were evaluated. We did not consider reviews, congress abstracts, or unpublished results. The references of the studies obtained were also examined to identify additional reports. We included all cases where a clear baseline diagnosis was made and where the onset of DM/PM was recorded after the use of anti-TNF-*α* agents (etanercept, lenercept, adalimumab, infliximab, lenercept, golimumab, or certolizumab).

*Data Extraction and Management*. The relevant study information was extracted (by AMB, one of the reviewers) into a Microsoft Excel database. Variables extracted included the following: age, sex, baseline disease, comorbidities, duration of illness until anti-TNF-*α* therapy initiation, anti-TNF-*α* treatment until DM/PM onset (drug, duration and, dosage), autoantibodies before and after anti-TNF-*α* therapy, concomitant treatments (drug, duration, and dosage) during anti-TNF-*α* therapy, improvement after withdrawal from anti-TNF-*α* therapy (yes, no, or partial), treatment received for DM/PM, complications, and outcomes. 

*Risk of Bias Assessment. *Two reviewers (AMB and CM) assessed the risk of selection biases to ensure that the criteria for diagnosis of the baseline disease and the new onset DM/PM were consistent and followed worldwide definitions to each disease.

## 3. Results 

We retrieved 13 publications describing a total of 20 patients who were treated with anti-TNF-*α* agents in the setting of dermatological or rheumatologic conditions outside DM and PM, between the years 2003 and 2012 [14–25]. We found 12 publications regarding the new onset of DM or PM in patients treated with anti-TNF-*α* agents for RA (17 cases), Crohn's disease (1 case), ankylosing spondilytis (AS, one case), and seronegative arthritis with a familiar history of psoriasis (1 case) [14–25].

Twenty patients received 21 cycles of therapy with TNF-*α* blockers. Nineteen patients experienced a new onset of DM, and one patient that received both adalimumab and infliximab experienced both a new onset and an exacerbation of DM [14–25]. In 7 cases the patients received etanercept, in 7 cases infliximab, in 5 cases adalimumab, and in two cases lenercept [14–25].

Twelve patients were female and 3 patients were male; in 5 cases sex was not reported [14–25]. The mean age at DM/PM diagnosis was 47.9 years (range: 29–57 years). The mean duration of the baseline illness until anti-TNF-*α* therapy initiation was 11.5 years (range: 4 months–33 years). The mean duration of the anti-TNF-*α* therapy until DM/PM onset was 11.7 months (range: 2 weeks–34 months) [14–25].

Regarding the presence of autoantibodies in serum before the initiation of anti-TNF-*α* therapies, in 3 patients antinuclear antibodies (ANA) were reported as negative (two cases of RA and one case of AS) in 6 patients as positive, and in 11 cases ANA were not available [14–25]. Between the 6 patients with positive ANA, one patient was also positive for anti-DNA-ds antibodies and three patients for anti-Jo-1 antibodies (see Table 1) [14, 19, 24].

After the initiation of the TNF-*α* blockers, in 5 patients the ANA-titers remained unchanged (in one patient the titer remained negative and in 4 cases the titer did not change); in 2 patients the titer increased and in 4 patients the ANA became positive. Anti-Jo-1 antibodies became newly positive in one patient after anti-TNF-*α* therapy introduction and in two patients other antisynthetase antibodies as anti PL-7 and anti PL-12 were reported as positive (see Tables 1 and 2) [14–25].

Regarding the 18 patients reported with arthritis and new onset of DM/PM, 17 cases had a baseline diagnosis of RA and one case was diagnosed as seronegative arthritis with a familiar history of psoriasis. Between the 17 cases of RA, in 7 patients there was incomplete information regarding duration of the baseline illness until anti-TNF-*α* initiation, autoantibodies status pre-TNF-*α* blocker therapy, and clinical and radiological description of the articular manifestations. In the remaining 10 patients with baseline diagnosis of RA, only in three cases the illness was present for less than one year (4, 6 and, 12 months) and the duration of the anti-TNF-*α* therapy was inferior to 9 months (2, 6, and 9 months); in one patient the ANA were positive before the initiation of adalimumab, and the titers increased after the introduction of therapy; in the second case, ANA and Jo-1 became positive after the initiation of etanercept and in the third case ANA were reported positive as well as anti-PL-12 [18, 19, 24]. In the first case, the patient was definitively diagnosed with undifferentiated overlap syndrome with features of polyarthritis, myositis, and scleroderma (dactylitis), possibly induced or exacerbated by adalimumab, and in the second patient a clear diagnosis of DM (with positive anti-DNA-ds and Jo-1 antibodies) with some features of seronegative polyarthritis (negative RF and anti-CCP antibodies) was made. In the third case, a definitive diagnosis of antisynthetase syndrome was attributed [18, 19, 24]. In the remaining 6 cases the previous diagnosis of RA was present for more than 3 years (3 to 33 years, mean: 19.2 years) and was confirmed by the clinical picture, by the radiological findings, and by the presence of RF and/or anti-CCP (2 patients). Between such patients, the previous autoimmune status was negative in two cases, not available in two cases, and positive for ANA in three cases, with three patients also positive for ant-Jo-1 antibodies. Between these 6 confirmed patients affected by RA, two became positive for ANA, one remained unchanged with positive ANA and Jo-1 antibodies, and one reported positive ANA one case with previous ANA positivity became also positive for Jo-1 antibodies and in one patient anti PL-7 antibodies were registered [14, 20, 22, 24].

Regarding patients with antisynthetase positive antibodies (anti-Jo-1, anti-PL-7, and anti-PL-12), the clinical findings regarding the presence of interstitial lung disease (ILD), Raynaud phenomenon, fever, and mechanical hand disease are summarized in Table 2.

Six patients received ant-TNF-*α* therapy as monotherapy; in 6 cases other concomitant drugs used were, prednisone (5 patients), methotrexate (5 patients) and tacrolimus (1 patient) (not available in 8 courses of therapy; see Table 1) [14–25].

Improvement of DM/PM after anti-TNF-*α* therapy suspension was recorded in 15 cases; only partial improvement was seen in one patient and there was no available information regarding outcome and/or complications in 5 cases. In all of the available cases with outcomes, therapy with other immunosuppressors was required to control the DM/PM flare (see Table 1). One fatal outcome was reported in a female patient that developed sepsis due to *Pneumocystis jiroveci *pneumonia, even after improvement from PM [20]. In two cases even after initial improvement, relapse of DM/PM was recorded in the absence of anti-TNF-*α* therapy [23, 24].

## 4. Discussion

Anti-TNF-*α* therapies are now commonly used in a variety of inflammatory conditions including RA, psoriasis, psoriatic arthritis, AS, and Crohn's disease. However, concerns have been raised regarding the safety profile of these agents [26].

In this systematic review we found 20 patients that received 21 cycles of anti-TNF-*α* therapy that developed DM/PM. Most of the patients (17 cases) were affected by RA [14–25]. The association between RA and DM/PM in a single patient seems to be infrequent and the real incidence of this overlapping is unknown [27–29]. The coincidence of myopathies and RA has been previously described in case series of patients (7 cases of DM and RA, 16 cases of PM and RA, and 15 cases of unspecified DM or PM associated to RA). However, no uniform criteria or detailed case records can be found regarding these cases and only 8 completely described case reports with clear overlap between RA and DM/PM (Brunasso and Massone, 2011, Martínez—Cordero et al., 2001, Nagashima et al., 2009, Nakajima et al., 2012) can be found in the literature [27–30]. Considering the rarity of the linkage between RA and inflammatory myopathies (DM/PM), most of the cases analyzed in the present study might not represent a sporadic association because of the history of introduction of an anti-TNF-*α* agent and the improvement after withdrawal of the drug. The causality between DM/PM and anti-TNF-*α* therapy in the cases examined in this systematic review can be described as not dose-related and time-delayed (range: 2 weeks to 34 months, mean: 12.7 months), being a typical finding of immunological adverse reactions [31, 32]. The association can be classified as probable and not confirmed in 19 patients (because rechallenge was not performed) and as confirmed in one patient (positive rechallenge with infliximab) [14–25, 31, 32]. It is worth noticing that a clear improvement after withdrawal was seen in almost all of the patients where information regarding followup and outcome was available (15 cycles of therapy) with the concomitant use of other immunosuppresssors [12].

It is important to consider that patients with DM may also have joint manifestations that can be misinterpreted as RA, even if these manifestations rarely cause joint deformities and destruction [2]. This might be the case with the two patients reported by Hall and Zimmermann and Liozon et al., as initially affected by RA, where the definite diagnosis after the introduction of the anti-TNF-*α* agent was undifferentiated overlap syndrome with features of polyarthritis, myositis, and scleroderma (dactylitis), possibly induced or exacerbated by adalimumab and in the second patient a clear diagnosis of DM probably without RA [18, 19]. In these two patients the role of the anti-TNF-*α* therapy might be only as accelerator/trigger of the disease onset [18, 19].

Drug induced myositis, usually associated with chronic use of corticosteroids and cloroquine in RA patients, can be another pitfall that might cause the incorrect diagnosis of overlapping RA and DM/PM [2, 30]. It is important to underline that there is no clear and confirmed relation between RA and DM/PM even if there is a significant increase in frequency of autoimmune diseases (included also RA) in first-degree relatives of patients affected by idiopathic inflammatory myopathies [2, 30]. This association between many autoimmune diseases can be explained by the fact that many disorders share genes that together act as polygenic risk factors for autoimmunity [33].

After the initiation of a TNF-*α* blocker in 5 of the 9 evaluable patients, the ANA-titer increased or became positive, as well the anti-Jo-1 antibodies that became positive in one patient and the anti PL-7 and anti-PL-12 that were reported as positive in another two cases. In 12 of the 13 patients, where reports regarding autoantibodies after anti-TNF-*α* introduction were available, the positivity was recorded. Only in one patient affected by AS, the autoantibodies profile remained negative after the introduction of infliximab and the development of PM [20]. The emergence of autoimmunity manifested as positive ANA, anti-DNA antibodies, and drug-induced lupus during anti TNF-*α* therapy has been widely documented [34, 35]. Skin manifestations such as purpura and photosensitivity in the context of autoimmunity are a well-known class effect of anti-TNF-*α* therapies, mostly infliximab rather than etanercept, with a frequency of autoantibodies up to 50% for ANA and 15% for anti-DNA antibodies [34, 35].

There is a specific subset of patients affected by DM/PM, where the presence of antisynthetase antibodies (Jo-1, PL-7, PL-12, EJ, OK, KS, YRS, and Zo) with the addition of some specific clinical findings as, ILD and/or arthralgia/arthritis constitute a clinical entity called antisynthetase syndrome [36–38]. In such patients, other clinical findings such as fever, Raynaud's phenomenon, and mechanic's hands can be present [36–38].

Between the 17 patients identified in the present study affected by RA as baseline diagnosis, three patients were reported with positive anti-Jo-1 antibodies before to the initiation of these anti-TNF-*α* therapy, another patient became positive for this autoantibodies after treatment with TNF-*α* blockers (in the absence of previous ANA positivity), and another two cases were reported as positive for anti-PL-7 and PL-12 antibodies after the introduction of etanercept [14–25].

It has been postulated that patients with baseline diagnosis of RA but with positive antisynthetase antibodies can develop an anthisynthesase syndrome; this might be the case of the 6 patients with positive antisynthetase antibodies described herein. In particular, in the five cases described by Musial et al, Urata et al, Ishikawa et al. and Ishiguro et al, the association between RA and ILD was previous to the introduction of the anti-TNF-a therapy [14, 17, 24]. Probably these patients were affected by antisynthetase syndrome as baseline disease (in absence of myositis) and the onset of myositis (DM in one case and PM in four cases) was triggered by the initiation of TNF-*α* blocker [14, 24].

Anti-Jo-1 antibody is the most common and specific antibody in DM/PM and is detected in approximately 15–33% of patents [30]. Other myositis-specific antibodies are detected only in 3-4% of patients [30]. Nakajima et al. in 2012 examined 12 cases of DM/PM proceeded by RA [30]. It is worth noticing that in 8 of such 12 cases, the criteria for the diagnosis of antisynthetase syndrome were present (positive antisynthetase antibodies, interstitial lung disease, and/or DM or PM, and/or arthritis) [30]. According to the authors, the fact that patients presented with erosive arthritis was an exclusion criteria for the diagnosis of antisynthetase syndrome [30]. Interestingly, there is evidence of erosive arthritis in patients with antisynthetase syndrome, and it has also been demonstrated that anti-CCP antibodies are markers of erosive arthritis in antisynthetase syndrome [39–42]. RA-like arthritis may be present in patients with antisynthetase syndrome (with anti-Jo-1 positive antibodies) independent of the occurrence of myositis, as suggested by Cavagna et al. [42]. In the 6 patients identified in the present study with positive antisynthetase antibodies, there was evidence of erosive arthritis in all of them and anti-CCP antibodies were positive in three cases, negative in one patient, and not available in the other two cases.

Between the 20 cases examined herein, one developed a subset of DM/PM called antisynthetase syndrome after the introduction of anti-TNF-*α* therapy. In the other five patients (three with previous positive anti-Jo-1 antibodies, one with anti-PL-7, and another one with anti PL-12) the emergence of PM/DM and the aggravation of the clinical picture previously compatible with RA associated with interstitial lung disease was compatible with a full development of an antisynthetase syndrome, probably unmasked by the use of anti-TNF-*α* agents. Interestingly, anti-TNF-*α* therapy has been associated also with the new onset or exacerbation of ILD mainly in patients affected by RA, being the TNF-*α*, a vital cytokine implicated in the development of pulmonary fibrosis [43, 44].

The use of anti-TNF-*α* therapies has been postulated in the treatment of DM/PM, in particular the use of etanercept as steroid-sparing agent [10–12]. Mainly case reports and series descriptions have been conducted; only one RCT is retrievable and larger studies are not available [11]. The muscle study group published in 2011, a randomized, double-blind, placebo-controlled trial evaluating the use of etanercept (50 mg subcutaneously weekly) for 52 weeks in DM affected patients [11]. The authors reported that there were no significant differences in adverse event rates between treatment-groups and that 5 of 11 (45.45%) etanercept treated patients successfully weaned off prednisone in contrast to all 5 patients on the placebo group that failed the prednisone withdrawal schedule (median time to treatment failure: 358 days) [11]. The authors concluded that there are no major safety concerns regarding the use of etanercept in DM, and a steroid-sparing effect deserves further investigation [11]. The results of this RCT were not conclusive, because of the small number of patients enrolled and because the efficacy was not even in the whole spectrum of DM: skin and muscle compartments (5 patients receiving etanercept experienced worsened skin rash and one case even improved after withdrawal) [12]. There are approximately 56 other patients and 63 courses of therapy reported in the literature regarding the use of anti-TNF-*α* agents in patients affected by DM/PM or juvenile DM [45–59]. In 53% of those cases (34 out of 63 courses of anti-TNF-*α* therapy), an improvement of the disease was recorded [45–59].

The paradoxical onset of DM/PM in patients treated with anti-TNF-*α* blockers is in conflict with the positive therapeutic effect previously mentioned of such agents [45–59]. The mechanism underlying this paradoxical phenomenon remains elusive, but the increased production of IFN-*γ* after TNF-*α* blockage might play a role, as IFN-*γ* is a key element in the induction of DM/PM [60]. The onset of unexpected and antagonistic reactions associated with targeted therapies has been previously described not only during anti-TNF-*α* therapy but also with other biologicals such as efalizumab, rituximab, and abatacept [60]. One of the most typical examples of such paradoxical reactions regards the new onset of psoriasis in patients treated with anti-TNF-*α* therapy for chronic inflammatory diseases as RA, inflammatory bowel disease, and AS [60]. The onset of unexpected and antagonistic reactions associated with these targeted therapies reveals the complexity of the immunogenetic pathways involved in human disease [60, 61].

TNF-*α* blockage may induce autoimmune phenomena in individuals with some genetic background as confirmed by the onset of autoantibodies (50% of antinuclear antibodies and 15% of anti-DNA antibodies), drug induced lupus, vasculitis, antiphospholipid syndrome, and other autoimmune entities [21, 49, 50, 60, 61]. Anti-TNF-*α* therapy inhibits the cytotoxic T lymphocyte response that would normally suppress the autoreactive B-cell response, promoting humoral autoimmunity and increasing the type-I interferon system, that has been implicated in the pathogenesis of DM and PM [22, 29, 39, 51]. The accumulation of apoptotic cells and the release of antigenic particles might stimulate autoimmunity [12]. The increased infections-rate in patients treated with anti-TNF-*α* drugs may lead to polyclonal B lymphocyte activation and autoantibody production [12]. The blockage of TNF-*α* causes the exacerbation or prolongation of preexisting autoimmune diseases, such as multiple sclerosis [12, 62].

This systematic review presents clear limitations associated with the small number of patients, the retrospective design, and the incomplete information data in many reports. The strength of our work is to summarize the information published about the new onset of DM/PM during anti-TNF-*α* therapies, a particularly interesting fact in the daily clinical routine because there is evidence for anti-TNF-*α* therapies (case series and one RCT) and many physicians used such molecules as steroid sparing agents in patients affected by DM/PM [13, 45–59].

In conclusion, the emergence of DM/PM and a specific subset of such diseases as the antisynthetase syndrome seem to be associated with the use of anti-TNF-*α* agents, especially in patients with chronic inflammatory diseases (mainly RA but also AS and Crohn's disease) and with positive autoantibodies before therapy initiation. In particular, physicians should pay attention to patients affected by RA with positive antisynthetase antibodies (in particular anti-Jo-1 antibodies) and/or history of ILD. In those cases the use of the TNF-*α* blocking agents may trigger the onset of PM, DM, and antisynthetase syndrome or may aggravate or trigger the lung disease.

## Figures and Tables

**Table 1:** Clinical characteristics of reported patients that developed polymyositis (PM)/dermatomyositis (DM) during anti-TNF-*α* therapy.

First author, year	Number of patients (age, sex)	Baseline diagnosis	Duration of illness until anti-TNF-*α* initiation	Anti-TNF-*α* therapy	Duration of anti-TNF-*α* therapy until diagnosis of DM/PM	Concomitant therapy during anti-TNF-*α* treatment	Autoantibodies before anti-TNF-*α* therapy	Autoantibodies after anti-TNF-*α* therapy	Improvement after withdrawal of anti-TNF-*α* therapy, treatment, and outcome
Musial, 2003 [14]	1 (52 y, F)	RA	20 y	Infliximab	30 mo	Pred + MTX	ANA 1: 320 dsDNA: neg Jo-1: pos	ANA 1: 320 dsDNA 1: 20 Jo-1: pos	Yes, corticosteroids

Flendrie, 2003 [15]	1 (NA)	RA	NA	Infliximab	NA	NA	NA	NA	NA

Flendrie, 2005 [16]	1 (52 y, F)	RA	NA	Lenercept	2.5 mo	NA	NA	NA	Yes, NA

Urata, 2006 [17]	1 (52 y, F)	RA + pulmonary fibrosis	33 y	Infliximab	9 mo	Yes (pred + MTX) but only for 6 weeks	ANA 1: 640 dsDNA: neg Jo-1: pos	ANA 1: 640 dsDNA: neg Jo-1: pos	Yes, corticosteroids

Hall, 2006 [18]	1 (44 y, F)	Seronegative RA	1 y	Etanercept	6 mo	MTX	ANA: neg Anti-CCP: neg Jo-1: neg	ANA 1: 640 dsDNA: NA Jo-1: pos	Yes, corticosteroids

Liozon, 2007 [19]	1 (47 y, F)	RA	6 mo	Adalimumab	9 mo	Pred (for 3 mo) and MTX	ANA 1: 640 dsDNA: pos Jo-1: neg Anti-CCP: pos	ANA 1: 2560 dsDNA: pos Jo-1: NA Anti-PM-Scl: pos	Yes, corticosteroids

Kiltz, 2008 [20]	2 pts (46 y, M)	AS	17 y	Infliximab	6 mo	NA	ANA: neg	ANA: neg	Yes, corticosteroids
	(57 y, F)	RA	26 y	Etanercept	30 mo	No	ANA 1: 160	ANA 1: 2560	Yes, pred + cyclophosphamide, but fatal outcome sepsis due to *Pneumocystis jiroveci* (carinii) pneumonia

Ramos-Casals, 2008 [21]	4 pts (NA)	RA	NA	Infliximab, etanercept, lenercept	NA	NA	NA	NA	NA

Brunasso, 2010 [22]	1 (45 y, F)	RA	13 y	Adalimumab	34 mo	No	ANA: neg	ANA 1: 320 Jo-1: neg	Yes, corticosteroids

Klein, 2010 [23]	3 pts (40 y, F)	RA	NA	Etanercept	2 y	No	NA	ANA: pos dsDNA: neg Jo-1: neg	Partial improvement, corticosteroids, recurrence after 8 mo
	(29 y, F)	Seronegative arthritis with familiar history of psoriasis	NA	Adalimumab	3 mo	MTX	NA	ANA 1: 640 dsDNA: neg Jo-1: neg	Yes, corticosteroids + methotrexate + azathioprine + quinacrine
	(51 y, F)	RA	NA	Adalimumab	2 mo	No	NA	NA	Yes, corticosteroids

Ishikawa, 2011 [24]	1 (52 y, F)	RA + NSIP	3 y	Etanercept	2 mo	No	ANA: pos dsDNA: neg Jo-1: pos Anti-CCP: pos	ANA 1: 320 dsDNA: neg Jo-1: pos	Yes, corticosteroids, but relapse of PM after 2 weeks

Ishikawa, 2011 [24]	1 (52 y, M)	RA + ILD	12 y	Etanercept	26 mo	NA	NA Anti-PL-7	ANA: neg	Yes, corticosteroids

Ishikawa, 2011 [24]	1 (63 y, F)	RA + NSIP	4 mo	Etanercept	2 mo	Tacrolimus + pred	Anti-CCP: pos ANA: NA	ANA 1: 320 anti-PL-12: pos	Yes, corticosteroids

Riolo, 2012 [25]	1 (36 y, M)	Crohn's disease	1 y	Adalimumab	2 weeks	No	ANA 1: 640 dsDNA: neg Anti-U1 RNP: pos	ANA 1: 640 dsDNA: neg Anti-U1 RNP: pos	Yes, corticosteroids + methotrexate
				Infliximab	1 mo	Pred + MTX	ANA 1: 640 dsDNA: neg Anti-U1 RNP: pos	ANA 1: 640 dsDNA: neg Anti-U1 RNP: pos	Yes, corticosteroids + methotrexate

DM: dermatomyositis, PM: polymyositis, y: years, F: female, M: male, RA: rheumatoid arthritis, mo: months, ANA: antinuclear antibodies, neg: negative, pos: positive, NA: not available, pts: patients, AS: ankylosing spondilytis, NSIP: nonspecific interstitial pneumonia, ILD: interstitial lung disease.

**Table 2:** Characteristics of patients reported regarding diagnostic parameters for antisynthetase syndrome.

Author, year	Antisynthetase antibodies pre-TNF-*α*	Antisynthetase antibodies after anti-TNF-*α*	Myositis	ILD	Arthritis	Raynaud's phenomenon	Fever	Mechanics hand disease
Ishikawa et al., 2011 [24]	NA	Anti-PL-12	Present/PM	Present	Present	Absent	Present	Absent
Ishikawa et al., 2011 [24]	NA	Anti-PL-7	Present/DM	Present	Present	Absent	NA	NA
Ishikawa et al., 2011 [24]	Jo-1	Jo-1	Present/PM	Present	Present	Absent	Absent	Absent
Hall and Zimmermann, 2006 [18]	Negative	Jo-1	Present/DM	Present	Present	Absent	Absent	Absent
Urata et al., 2006 [17]	Jo-1	Jo-1	Present/PM	Present	Present	Absent	Absent	Absent
Musial et al., 2003 [14]	Jo-1	Jo-1	Present/PM	Present	Present	Absent	Present	Absent

ILD: interstitial lung disease, PM: polymyositis, DM: dermatomyositis, NA: not available.
